# Over a thousand-fold enhancement of the spontaneous emission rate for stable core−shell perovskite quantum dots through coupling with novel plasmonic nanogaps

**DOI:** 10.1515/nanoph-2023-0751

**Published:** 2024-01-30

**Authors:** Vanna Chrismas Silalahi, Dokyum Kim, Minjun Kim, Samir Adhikari, Seongmoon Jun, Yong-Hoon Cho, Donghan Lee, Chang-Lyoul Lee, Yudong Jang

**Affiliations:** Department of Physics, Chungnam National University, Daejeon 34134, Republic of Korea; Advanced Photonics Research Institute (APRI), Gwangju Institute of Science and Technology (GIST), Gwangju 61005, Republic of Korea; Department of Physics and KI for the NanoCentury, Korea Advanced Institute of Science and Technology (KAIST), Daejeon 34141, Republic of Korea; Institute of Quantum Systems (IQS), Chungnam National University, Daejeon 34134, Republic of Korea

**Keywords:** emission rate, enhancement, core−shell perovskite quantum dots, stability, nanogap

## Abstract

High Purcell enhancement structures and stable emitters are essential prerequisites for the successful development of novel fast-operating active devices. Furthermore, a uniform enhancement of the spontaneous emission rate is critical for practical applications. Despite considerable efforts being made to meet these requirements, achieving them still remains a challenging task. In this work, we demonstrate that placing stable core−shell perovskite quantum dots (PQDs) in the nanogap region of hole/sphere-based nanogap structures (HSNGs) can enhance the spontaneous emission rate by more than a thousand-fold (up to a factor of ∼1080) compared to PQDs in solution. This enhancement factor is the highest value reported using PQDs, exceeding previously reported values by two orders of magnitude. Notably, the enhancement factor of the emission rate in the HSNG maintains large values across the samples, with values ranging from ∼690 to ∼1080. Furthermore, the structural stabilities of the PQDs are remarkably enhanced with the incorporation of SiO_2_ shells, which is validated by monitoring the changes in photoluminescence intensities over time during continuous laser exposure. As a result, the HSNG with stable core−shell PQDs offers great potential for fast optical device applications that require high performance and long-term operational stability.

## 1 Introduction

A fast carrier lifetime of emitters is desirable for fast operation of various optical devices. However, typical emitters such as molecules, quantum dots (QDs), and carbon nanotubes have rather long lifetimes (in normal conditions), ranging from around 1 to 10 ns [1], [2], [3], which restricts the operation speed of devices to less than the order of gigahertz. The spontaneous emission rate of an emitter is significantly enhanced when it is placed in strong electric field formed by manipulation of the surrounding environment, known as the Purcell effect [4]. One way to increase the radiative decay rate of emitters using the Purcell effect is to place them at the antinodes of high-*Q* dielectric cavities such as micropillars [5], [6], [7], microdisks [8], and photonic crystal [9]. This configuration allows for increased interaction between the emitter and the cavity mode, resulting in an enhanced radiative decay rate. However, it is very difficult to achieve Purcell factor (PF) values higher than 100 in these dielectric cavities [5], [6], [7], [8], [9]. Moreover, these high-*Q* cavities have a very narrow-band cavity mode (<1 nm), making it difficult to match the emission wavelength to the cavity mode.

To compensate for this, a proper design of the plasmonic nanostructure can produce hotspots, in which the very strong electric field is spatially confined. Placing the emitters in hotspots can strongly increase the emission rate. Furthermore, plasmonic nanostructures exhibit broad localized surface plasmon resonance (LSPR) modes, which facilitate convenient wavelength matching with emitters. Accordingly, various plasmonic structures including nanocubes [1], [2], nanocavities [3], [10], nanogaps [11], and bowtie antennas [12] have demonstrated a strong enhancement of the emission rate. Recently, the combination of plasmonic nanopatch antennas (NPAs) and CdSe/ZnS QDs has been demonstrated to strongly enhance the spontaneous emission rate, with PFs as high as 880 [1]. However, the enhancement factor varies under the NPA, ranging from 190 to 880, due to the random spatial distribution of QDs and a huge variation in the electric field across the nanocube. Similarly, silicon nanoparticles on a metal film (SiNPoM) with QDs in the gap showed a large variation in emission enhancement ranging from 69 to 826 [10]. Recently, many studies have reported Purcell-enhanced emission of perovskite QDs (PQDs) through the utilization of plasmonic nanostructures, but the enhancement factors did not exceed about 10 [13], [14], [15], [16]. In addition, the enhancement factors also showed low reproducibility. Therefore, a novel plasmonic platform with high reproducibility and uniformity is still in high demand for system applications.

High-quality emitters are essential components for ensuring optimal performance of photonic devices. Recently, PQDs have gained a lot of attention as scalable and color-tunable single emitters. Specifically, cesium lead halide PQDs have the lowest excited state in the optically bright exciton state, which results in a fast carrier lifetime [17]. Furthermore, PQDs have high absorption cross-sections, wide and easy spectral tunability, and high photoluminescence (PL) quantum yields (PLQYs) of 60–90 % [18], [19], [20], [21]. Besides, single-photon emission from a single PQD was recently demonstrated [19], [20], revealing the potential of PQDs as single-photon sources. Nevertheless, poor structural stabilities due to their ionic nature against moisture, polar solvents, heat, and oxygen have restricted their practical applications [21]. Fast structural degradation of CsPbI_3_ QDs under moisture and/or air exposure has been observed, where all PQDs became non-luminescent within 1 h [21]. In recent studies, the structural stabilities of PQDs were shown to be significantly enhanced by introducing ultrathin SiO_2_ shells (∼2 nm), making a core−shell structure. Core−shell CsPbBr_3_@SiO_2_ QDs can be successfully prepared using a modified hot-injection method with 3-aminopropyl-triethoxysilane (APTES) [22]. Note that, high structural stabilities of core−shell PQDs contribute high experimental reproducibility.

In this work, we introduce a novel architecture called as the hole/sphere–based plasmonic nanogap structure (HSNG) and demonstrate that the coupling of structurally stable core−shell CsPb(Br_0.2_I_0.8_)_3_@SiO_2_ QDs with HSNG antenna enhances the spontaneous emission rate of PQDs by more than a thousand-fold, exceeding previously reported values using PQDs by two orders of magnitude. To the best of our knowledge, it is the first study to combine core−shell PQDs with plasmonic nanogap antennas, which has not been reported to date. Moreover, the enhancement factor of over 1000 times (∼1080) is the highest value reported using PQDs. The enhancement factor for the spontaneous emission rate of PQDs within the HSNG ranges from ∼690 to ∼1080 and is quite consistent and highly reproducible across samples, due to relatively uniform enhancement of the average electric field within the nanogap. In addition, high structural stabilities of core−shell PQDs also enables high experimental reproducibility, which was confirmed by the results showing little change in the photoluminescence intensity of PQDs under continuous laser exposure.

## 2 Methods

### 2.1 Fabrication of the hole/sphere-based plasmonic nanogap structure (HSNG)

A Si wafer was cleaned with acetone at 180 °C for 10 min, methanol at 180 °C for 10 min, and isopropyl alcohol (IPA) at room temperature (RT) for 5 min. The wafer was dried with N_2_ gas and put on a hot plate at 110 °C for 5 min. Then core−shell CsPb(Br_0.2_I_0.8_)_3_@SiO_2_ QDs were spin-coated onto the Si wafer at a speed of 6000 rpm for 50 s. To fabricate the nanogap substrate, a 5 × 5 mm^2^ Si wafer was first coated with a 5 nm thick layer of Cr followed by a 100 nm thick layer of Au using an electron beam evaporator. Next, a 20 nm thick layer of SiO_2_ was deposited onto the wafer at 350 °C using plasma-enhanced chemical vapor deposition. To create the gold nanoparticles that would form the nanogaps, a 10 nm thick layer of Au was deposited onto the SiO_2_-coated Si wafer using an electron beam evaporator. The Au nanoparticles were self-assembled by annealing the Au thin film substrate at 820 °C for 2 min. Wet etching was then carried out to form the nanogaps. The SiO_2_ layer was selectively etched using an NH_4_HF_2_:HF (60:1) solution for 120 s. Finally, a 45 nm thick layer of Au was deposited using an electron beam evaporator, completing the formation of the nanogap substrate.

### 2.2 Preparation of core−shell PQDs

To prepare Cs-oleate, 0.6 mmol of Cs_2_CO_3_ (99 %, Sigma–Aldrich), 0.7 mL of oleic acid (OA, 90 %, Sigma–Aldrich), and 8 mL of 1-octadecene (ODE, 90 %, Sigma–Aldrich) were added to a 25 mL two-neck flask and degassed under vacuum at 100 °C for 30 min. Then 0.07 mmol of PbBr_2_ (98 %, Sigma–Aldrich) and 0.27 mmol of PbI_2_ (99.999 %, Sigma–Aldrich) were added to a 100 mL two-neck flask with 15 mL of ODE and degassed under vacuum at 100 °C for 30 min. After degassing, the inside of the two-neck flask was converted from a vacuum to a N_2_ atmosphere, and then 2 mL of OA, 0.4 mL of oleylamine (OAm, 70 %, Sigma–Aldrich), and 1 mL of 3-aminopropyltriethoxysilane (APTES, 98 %, Sigma–Aldrich) were added to dissolve the Pb precursor. Then the temperature of the Pb-pot was raised to 165 °C, and the prepared Cs-oleate (preheated to 150 °C) was quickly injected. The synthesized solution was quenched in an ice bath after ∼10 s reaction. To collect the core−shell CsPb(Br_0.2_I_0.8_)_3_@SiO_2_ QDs, the supernatant solution was first centrifuged with acetone at 8,000 rpm for 3 min, and then, the precipitates were centrifuged with methyl acetate at 15,000 rpm for 5 min. Finally, the precipitates were dispersed in octane.

### 2.3 Optical measurements

For the sample preparation, 2 μL of PQDs dispersed in octane were dropped onto the nanogap surface using a pipette, followed by drying at RT for about 1 h. The optical properties of the samples were investigated by performing PL measurements at RT. The samples were excited with a continuous wave laser of 405 nm at an excitation density of 3.9 W/cm^2^. The PL signal was collected using a collection lens and spectrometer (SPEX 1805) with a TE-cooled charge-coupled device (CCD) array. A 532 nm long pass filter (Semrock) was used to remove the scattered laser light. The PL emission rates of the PQDs were measured using a time-correlated single-photon counting (TCSPC) technique and a time-resolved PL (TR-PL) technique with a streak camera. A pulse laser with a wavelength of 405 nm, a repetition rate of 20 MHz, and a pulse width of <40 ps (Alphalas GmbH) was used to excite the PQDs. The emitted photons were detected using a single-photon detector (ID100, ID Quantique), and the resulting TR-PL signals were collected and processed using a TCSPC module (CD900, Edinburgh Instruments). During the TCSPC measurements, the signal emitted from the PQDs was selected using a bandpass filter (bandwidth of ∼10 nm) with a center wavelength at each PL peak. In the TR-PL measurements, the samples were excited using a second harmonic generated Ti:Sapphire laser with a wavelength of 405 nm. The repetition rate and pulse width of the laser were 80 MHz and ∼200 fs, respectively. The TR-PL signal was filtered using a spectrometer (model C10627-01, Hamamatsu Photonics) to isolate the PL peaks before detection. Then the signal was detected with a streak camera from Hamamatsu Photonics (C7700-01). The system’s instrument response function was measured to be as low as ∼20 ps.

### 2.4 Finite-difference time-domain (FDTD) simulation

A commercial software package (Ansys Lumerical FDTD Solutions) was used to perform simulations using the finite-difference time-domain (FDTD) method. To obtain the localized surface plasmon resonance of the nanogap devices, we used the so-called total-field/scattered-field method. In this method, a planewave normally incident on the nanogap structure and the scattered field out of the structure are monitored. The mesh size around the nanogap structure was uniformly set to 1 nm in all directions. Symmetric and antisymmetric boundary conditions were set in the in-plane directions. The boundary condition in the *z*-direction was set as a perfectly matched layer (PML). For the calculation of the Purcell factor, a dipole was placed at the center of the nanogap region with its polarization aligned normal to the facing Au surfaces. The mesh sizes around the dipole were 1 nm in all directions. The Purcell factor was calculated as the ratio of total transmission through a transmission box (5.5 nm × 5 nm × 5 nm) surrounding the dipole to that for a dipole in free space. The PML boundary condition was set in all directions. The dielectric functions used for SiO_2_ and Si were obtained from Palik [23], and that for Au was obtained from Johnson and Christy [24].

## 3 Results and discussions

### 3.1 Hole/sphere-based plasmonic nanogap structure (HSNG)

**Figure 1:**
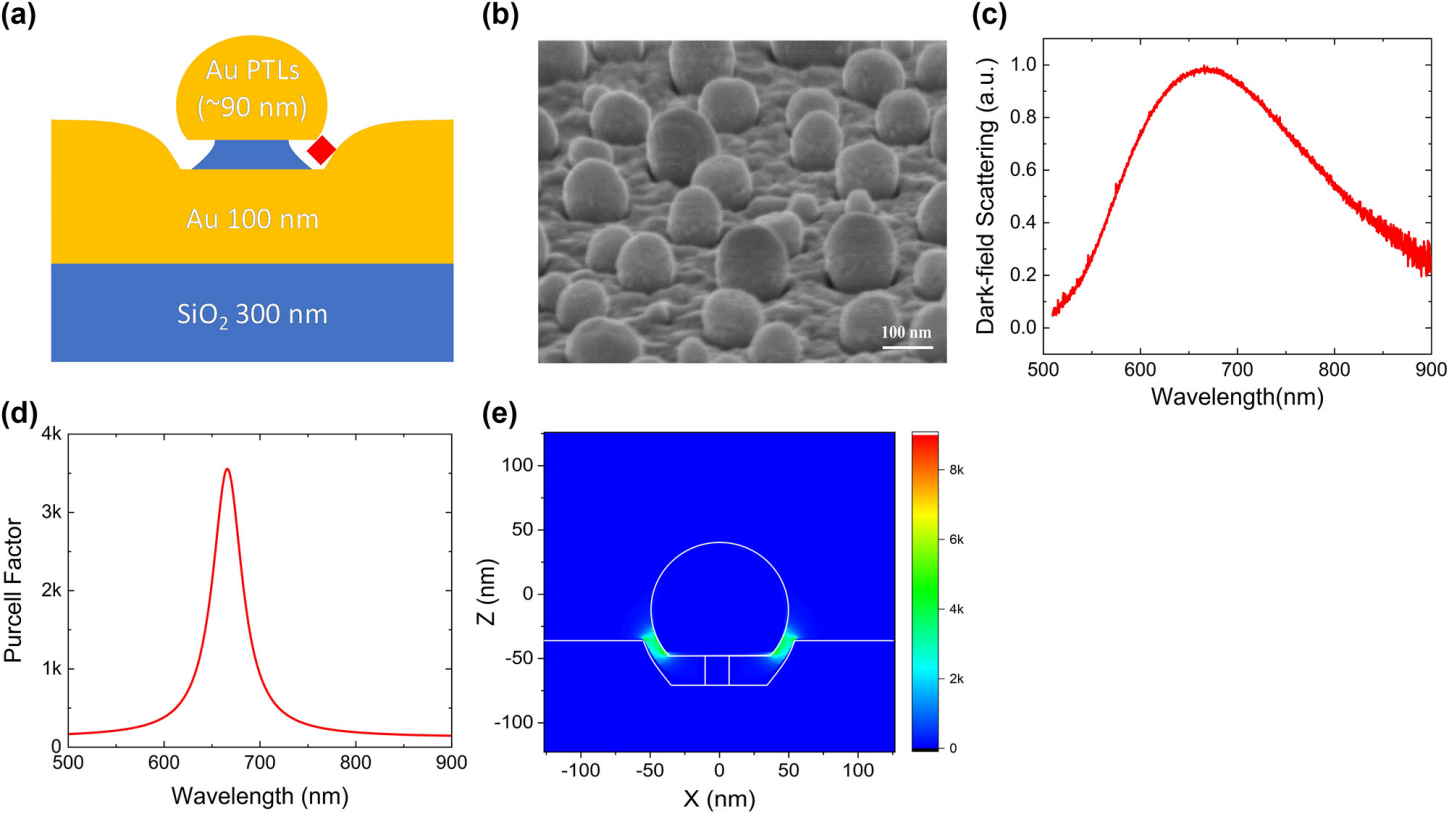
The HSNG structure. (a) Schematic illustration of the HSNG. (b) SEM image (tilted-view) of the HSNG. (c) Dark-field scattering spectrum of the HSNG. (d) PF spectrum of the HSNG. (e) Electric field intensity distribution, |*E* (*X*, 0, *Z*)|^2^/|*E*_0_|^2^, in the HSNG at *λ* = 666 nm (*D* = 95 nm, *s* = 10 nm).

The HSNG consists of a truncated gold sphere and a flat gold surface placed in proximity on a SiO_2_ substrate, as shown in Figure 1(a). The LSPR of the HSNG can be precisely tuned by adjusting the size of the spheres and the nanogap distance during the fabrication processes, which is a notable advantage of the HSNG. A SEM image of the fabricated HSNG is shown in Figure 1(b). Nanogaps are clearly formed as viewed in the tilted direction. The average diameter (*D*) of the truncated Au spheres is 87 ± 28 nm, and the size (*s*) of the nanogaps is estimated to be 13 ± 3 nm. The LSPR spectrum of the HSNG is obtained *via* dark-field scattering measurement, showing resonance at ∼660 nm (Figure 1(c)).

The FDTD simulation confirms the performance of the HSNG with *D* = 95 nm and *s* = 10 nm according to the SEM data. During the FDTD simulation, the structural dimensions were chosen to produce LSPR around 660 nm, which falls within the measured dimension range. The calculated PF spectrum of the HSNG is displayed in Figure 1(d). The maximum PF value is about 3500 at a resonance wavelength of 666 nm. The electric field distribution of the HSNG at the resonance wavelength is shown in Figure 1(e). The strong electric field intensity, normalized to that in vacuum, is well localized within the nanogap region with the maximum value exceeding 3000. Furthermore, the electric field intensity within the nanogap is rather uniformly distributed, implying that the emitter experiences a consistently strong Purcell enhancement when trapped in the nanogap. This is in stark contrast to previous report employing NPA structures, which showed a large variation in PF values in the nanogap region [1]. The HSNG demonstrates remarkably strong and uniform plasmonic Purcell enhancement due to its strong field confinement, uniform field distribution in the nanogap region, and controllable resonance. Note that very strong and uniform surface enhanced Raman scattering was recently reported using a geometry similar to the present structure [25].

### 3.2 Characteristics of core−shell perovskite QDs with high structural stabilities

**Figure 2:**
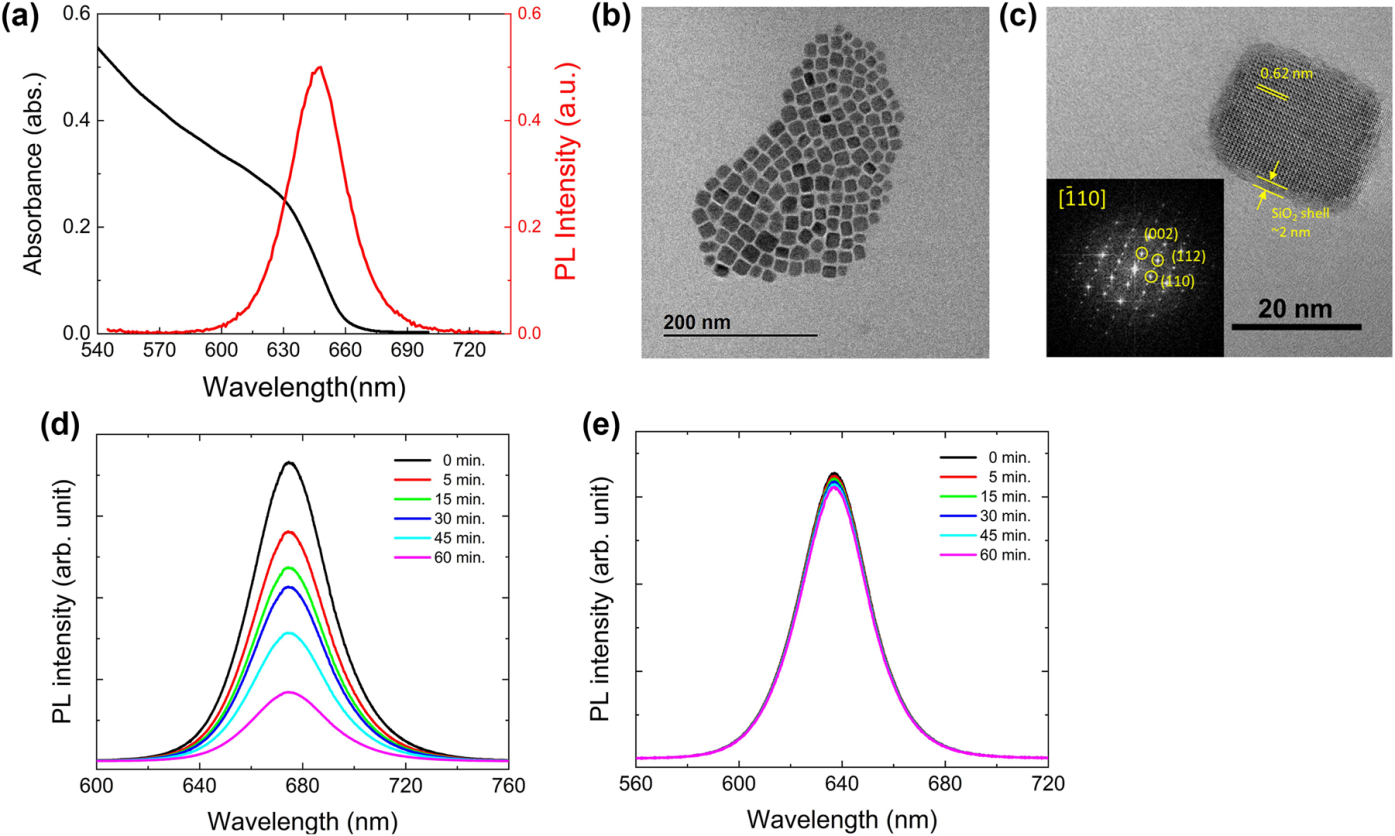
Core−shell CsPb(Br_0.2_I_0.8_)_3_@SiO_2_ QDs. (a) Absorption (black) and PL (red) spectrum of core−shell CsPb(Br_0.2_I_0.8_)_3_@SiO_2_ QDs dispersed in octane. (b, c) TEM images of core−shell CsPb(Br_0.2_I_0.8_)_3_@SiO_2_ QDs. (d) The PL spectra of core−only CsPbI_3_ QDs on SiO_2_ substrate and (e) those of core−shell CsPb(Br_0.2_I_0.8_)_3_@SiO_2_ QDs on HSNG according to laser illumination time.

The PL emission of core−shell CsPb(Br_0.2_I_0.8_)_3_@SiO_2_ QDs in solution was determined to be 647 ± 3.6 nm with a full width at half maximum (FWHM) of ∼31 nm from PL measurement at RT, as shown in Figure 2(a). The absolute PLQY of core−shell CsPb(Br_0.2_I_0.8_)_3_@SiO_2_ QDs in solution was measured to be ∼72 %. As shown in Figures 2(b) and S1, the core−shell PQDs exhibited a cubic structure and had a relatively small average size distribution of 18.60 ± 3.55 nm. It can be seen in Figure 2(c) that the SiO_2_ shell was uniformly coated with an ultrathin thickness of ∼2 nm. The SiO_2_ shell reduces structural degradation from external stimuli, such as heat or moisture, and thus improves structural stabilities of PQDs. We investigated the effect of SiO_2_ coating on the optical stabilities of core−shell CsPb(Br_0.2_I_0.8_)_3_@SiO_2_ QDs by monitoring their PL intensity changes for 60 min under continuous wave laser excitation at a power density of ∼80 W/cm^2^. Figure 2(d) and (e) show PL spectra of core−only CsPbI_3_ QDs on SiO_2_ substrate and core−shell CsPb(Br_0.2_I_0.8_)_3_@SiO_2_ QDs on HSNG, respectively. The PL intensities of the core−only CsPbI_3_ QDs decreased by 50 % after 60 min, while the PL intensities of the core−shell CsPb(Br_0.2_I_0.8_)_3_@SiO_2_ QDs remained unchanged, indicating superior structural stabilities of core−shell PQDs. The SiO_2_ shell successfully acts as a protective layer for the core−shell CsPb(Br_0.2_I_0.8_)_3_@SiO_2_ QDs, preventing them from being damaged or degraded when exposed to continuous laser excitation.

### 3.3 PL enhancement of core−shell PQDs in the HSNG

**Figure 3:**
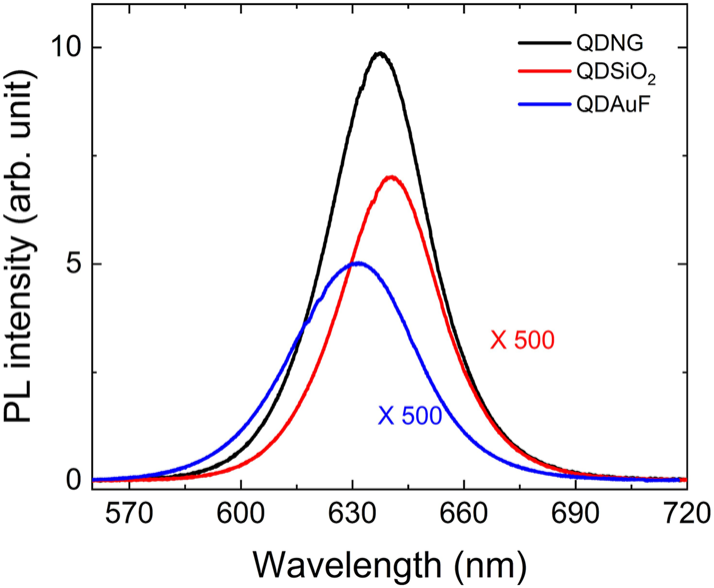
PL spectra of PQDs in HSNG (QDNG), on SiO_2_ substrate (QDSiO_2_), and on Au film (QDAuF) at RT. The PL intensities of QDSiO_2_ and QDAuF are multiplied by 500 to display them on the same scale as that of QDNG.

Enhanced PL intensities can provide significant advantages in device applications that require high efficiency and the detection of weak signals. The PL intensities of PQDs in the HSNG can be enhanced by increasing the spontaneous emission rate because it is directly proportional to the radiative decay rate. We compared the PL intensities of PQDs in three different structures at RT; PQDs spin-coated on SiO_2_ substrate (QDSiO_2_), PQDs spin-coated on Au film (QDAuF), and PQDs drop-casted in the HSNG (QDNG). The average density of the PQDs was adjusted to have 4–6 PQDs within a 100 nm × 100 nm area for the QDSiO_2_, QDAuF, and QDNG samples, which was confirmed from SEM images (Figure S2(a) and (b)). As shown in Figure 3, the PL intensity of QDNG is dramatically enhanced compared to that of QDSiO_2_ and QDAuF, being more than 500 times stronger than the PL intensities of the other samples. Moreover, the PL intensity of QDAuF is weaker than that of QDSiO_2_, indicating the presence of additional non-radiative processes due to Au-related loss [26]. The enhancement factor of the PL intensity of QDNG was calculated by taking the ratio of its spectral intensity to that of the reference sample (QDSiO_2_), resulting in a value of 711. The enhancement factor of the PL intensity of QDNG was measured at seven random positions on the sample and found to vary from 704 to 711, with an average value of 707 when compared to the PL intensity of QDSiO_2_.

Quantitative analysis of the PL enhancement of QDNG is complicated because the measured PL intensity is influenced not only by the PF but also by the geometry of the environment that determines the radiation pattern as well as by enhanced absorption. However, in this study, we expect that the Purcell effect is the dominant factor among them in the enhancement of the PL intensity of QDNG. The radiation pattern is not expected to have a significant influence on the enhancement factor. In our macro-PL measurement system, the collection angle of PL emission is only ±7°, corresponding to a numerical aperture of 0.125. To clarify this point, far-field ratios within the collection angle were calculated using FDTD simulation for QDNG and QDSiO_2_ at the wavelength of their PL peaks, and were found to be 0.016 and 0.020, respectively. In addition, the far-field ratio of QDSiO_2_ was calculated by taking the average of the values obtained from the dipole being polarized along the *x*, *y*, and *z* axes since the polarization of PQDs does not exhibit any specific directionality. The difference between the two ratios was approximately 1.3, which make a minor or negligible contribution to the enhancement of PL intensity in this case, considering that the PF of QDNG is two orders of magnitude higher than that of QDSiO_2_. Otherwise, absorption enhancement may not substantially contribute to the observed PL enhancement because the excitation wavelength used for PL measurement was 405 nm, which is significantly detuned from the resonance peak wavelength of the HSNG at 660 nm. Additionally, PQDs within the HSNG may not be efficiently excited compared to PQDs on SiO_2_ substrate and gold film due to their geometric positioning underneath gold nanospheres. Considering various conditions mentioned above, the PL enhancement of QDNG is expected to be further increased. In addition, no aggregation of PQDs on the substrate due to the low density of PQDs also contributes the enhanced PL intensity.

### 3.4 Enhancement of spontaneous emission rate in the HSNG

**Figure 4:**
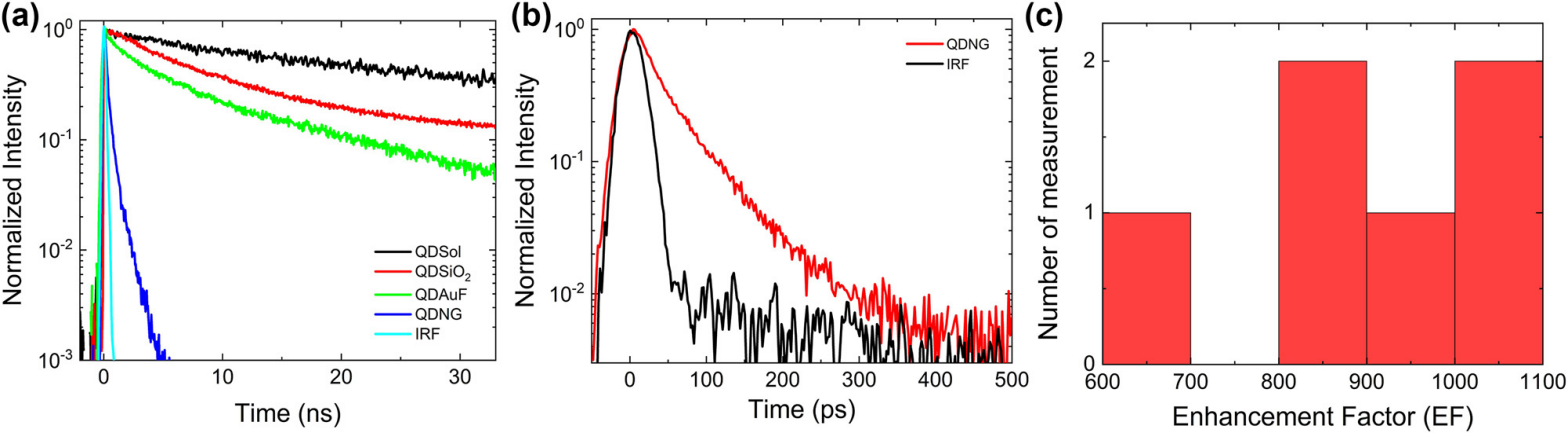
TR-PL properties of PQDs in the HSNG. (a) TR-PL of PQDs in solution (QDSol), QDSiO_2_, QDAuF, and QDNG measured by TCSPC at RT. (b) TR-PL of QDNG measured by a streak camera at RT. The instrument response function (IRF) is ∼20 ps. (c) Histogram of the enhancement factor (EF) distribution of the measured data.

The PF is defined as the ratio of the radiative decay rate of an emitter inside a cavity or plasmonic structure to its radiative decay rate in free space [4]. When a QD is placed in a region of very strong electromagnetic field localization, the local density of states increases, resulting in an enhanced photon emission rate. Figure 4(a) displays PL decay curves of PQDs in different environments obtained from time-correlated single-photon counting (TCSPC) measurements at RT. Results show that the PL decay of QDNG is much faster compared to those of QDs in solution (QDSol), QDSiO_2_, and QDAuF. The decay profiles of PQDs on different substrates can be fitted using a bi-exponential function: $A_{0}+A_{1}\exp\left(-t/\tau_{1}\right)+A_{2}\exp\left(-t/\tau_{2}\right)$ . In this equation, *A*_0_ represents the background signal, *A*_1_ and *A*_2_ represent the relative amplitudes, and *τ*_1_ and *τ*_2_ represent the decay time. Weighted average PL decay time (*τ*_*w*_) is calculated by considering the relative amplitudes of each time constant, defined as $\tau_{w}=\sum_{i}A_{i}\tau_{i}/\sum_{i}A_{i}$ [27]. The weighted average PL decay time (*τ*_*w*_) of QDNG is ∼0.2 ns, whereas for QDSol it is ∼67 ns. For QDSiO_2_, and QDAuF, *τ*_*w*_ is ∼8.8 ns and ∼3.3 ns, respectively. The shorter PL decay times of QDSiO_2_ and QDAuF, in comparison to PQDs in solution can be attributed to an increased non-radiative decay rate with these substrates. As shown in Figure 3, despite of its shorter lifetime, the PL intensity of QDAuF is comparable to that of QDSiO_2_, which can be attributed to the additional contribution of the reflected emission of PQDs from the Au film.

The first component (*τ*_1_) in the fitted PL decay time of QDNG is ∼100 ps, which is much shorter than the ∼200 ps instrument response function (IRF) of the TCSPC system. Therefore, to obtain a more accurate result of the TR-PL emission dynamics, we performed TR-PL measurements at RT with a streak camera that has an IRF of ∼20 ps. This allowed us to resolve the fast decay dynamics of the PQDs in the nanogaps with higher precision. Figure 4(b) shows the PL decay curve of QDNG together with the IRF. The PL decay is also fitted with two decay components, resulting in *τ*_1_ of ∼13 ps and *τ*_2_ of ∼125 ps and gives *τ*_*w*_ = 62 ps. The PL decay time of the PQDs is remarkably reduced in the HSNG, which is attributed to an enhancement of the spontaneous emission rate due to the strong field enhancement of the nanogaps.

The enhancement of the spontaneous emission rate of QDNG induced by a strong field in the nanogaps can be quantified by comparing the PL decay time of PQDs in solution to that of QDNG (*τ*_Sol_/*τ*_InNG_). The PL decay rate of QDNG significantly increases since PQDs in the HSNG experience a strong Purcell effect. The enhancement factor (EF) of the spontaneous emission rate of QDNG relative to that of PQDs in solution was determined to be 1080, as detailed below. The PF (*F*_*P*_) of PQDs in the HSNG can be calculated using the obtained EF. The relationship between the EF of QDNG relative to PQDs in solution and the PF can be expressed as follows:

(1) $$EF=\frac{\tau_{sol}}{\tau_{QDNG}}=\frac{\tau_{0}QY_{Sol}}{\left(\tau_{0}/PF\right)QY_{QDNG}}=\left(\frac{QY_{Sol}}{QY_{QDNG}}\right)F_{P}$$

Here, QY_Sol_ and QY_QDNG_ are the quantum yields of PQDs in solution and QDNG, respectively, and *τ*_0_ (=1/*R*_*r*_) represents the radiative recombination time in solution. QY_Sol_ and QY_QDNG_ are expressed using the following equations:

(2) $$\begin{array}{ll} QY_{Sol} & =R_{r}/\left(R_{r}+R_{nr}\right),\\ QY_{QDNG} & =F_{p}R_{r}/\left(F_{p}R_{r}+R_{nr}+R_{q}\right), \end{array}$$

where *R*_*r*_ and *R*_*nr*_ represent the radiative and non-radiative decay rate of PQDs in solution, respectively. *R*_*q*_ is an additional non-radiative decay rate related to metal. *R*_*r*_ and *R*_*nr*_ of the PQDs in solution can be calculated using the measured PL decay time (*τ*_sol_) and QY_Sol_ for PQDs in solution, and *R*_*q*_ can be calculated from the PL decay rate of QDAuF because that Au-related loss is the dominant non-radiative process in QDNG. In this way, PF can be obtained from the EF.

For the PQDs in solution, QY_Sol_ was found to be ∼72 % and the PL decay time ( $\tau_{w}^{\text{sol}})$ was ∼67 ns, allowing us to calculate the rates of radiative decay (*R*_*r*_) and non-radiative decay (*R*_*nr*_) as 0.0107 ns^−1^ and 0.0042 ns^−1^, respectively. *R*_*q*_ was calculated to be 0.29 ns^−1^. The EF of QDNG relative to PQDs in solution was thus determined to be 1080. By substituting these values into Eqs. (1) and (2), the calculated PF (=*τ*_0_/*τ*) of QDNG was determined to be approximately 1480. To the best of our knowledge, this is the highest value reported to date for PQDs. The quantum yield of QDNG (QY_QDNG_) approaches unity as the PF becomes extremely large, which is inferred from Eq. (2). Under such conditions, the loss of carriers through non-radiative processes becomes negligible, and the decay time of carriers is determined solely by the radiative emission rate. QY_QDNG_ is calculated to be 98.2 %. Thus, it can be inferred that the decay of carriers in the PQDs in the HSNG occurs primarily through radiative processes.

A histogram of the EF distribution is presented in Figure 4(c). The EF ranges from ∼690 to ∼1080, corresponding to a PF range of ∼935 to ∼1480. These results suggest that the PFs in the HSNG exhibit consistently high values over the sample, despite some degree of variation. High consistency of high PFs over the sample is attributed to the geometrical characteristics of the HSNG. Due to the formation of a strong electric field region, exhibiting an almost cylindrical symmetry along the periphery of the Au spheres, considerable enhancement of the spontaneous emission rate occurs irrespective of the precise placement of the PQDs within the nanogap area. Although the level of enhancement of each individual hole/sphere-nanogap in the overall HSNG varies, the average field enhancement within the excitation spot remains relatively uniform across all sample locations [25]. This results in the observed high PF values over the sample. The experimentally obtained PF of ∼1480 from the QDNG sample is the highest value among the reported results using PQDs to date. However, it is worth noting that there is a size mismatch between the nanogap distance of the HSNG (approximately 10 nm) and the size of the PQDs (∼18 nm), which hampers maximum enhancement. Besides, the PL peak of the PQDs deviates from the LSPR peak of the HSNG, which further weakens the enhancement. Therefore, we expect that there is still potential for further PF improvement by improving the alignment of the size and resonance PL wavelength.

## 4 Conclusions

In conclusion, we have realized a huge enhancement of the radiative decay rate from stable core−shell PQDs by incorporating them into a hole/sphere-based plasmonic nanogap structure (HSNG). Enhancement of the spontaneous emission rate of the PQDs in the HSNG ranged from 690 to 1080, resulting in Purcell factors ranging from 935 to 1480. Such values represent an unprecedented PF increase for PQDs, surpassing previously reported values by two orders of magnitude. Notably, the enhancement of the spontaneous emission rate in QDNG exhibited consistently high values across the sample due to relatively uniform average field enhancement within the excitation spot on the HSNG. Furthermore, excellent structural stabilities of the core−shell PQDs in HSNG ensure long-term stability and reliability of our system. Our findings suggest that the HSNG with stable core−shell PQDs has promising potential to serve as an excellent platform for highly stable and fast optical applications.

## Supplementary Material

Supplementary Material Details
