# Supplementary material for: Over a thousand-fold enhancement of the spontaneous emission rate for stable core−shell perovskite quantum dots through coupling with novel plasmonic nanogaps
Source: Nanophotonics. 2024 Jan 30;13(3):369–76. doi: 10.1515/nanoph-2023-0751 (PMC11501862; doi:10.1515/nanoph-2023-0751)
Supplement: Supplementary file 1 — Supplementary Material Details [file j_nanoph-2023-0751_suppl_001.docx]

Over a Thousand-Fold Enhancement of the Spontaneous Emission Rate of Stable Core−Shell Perovskite Quantum Dots through Coupling with Novel Plasmonic Nanogaps

Vanna Chrismas Silalahi^1,†^, Dokyum Kim^2,†^, Minjun Kim^1,†^, Samir Adhikari^1^, Seongmoon Jun^3^, Yong-Hoon Cho^3^, Donghan Lee^1,4^, Chang-Lyoul Lee^2,*^, and Yudong Jang^4,*^

^1^Department of Physics, Chungnam National University, Daejeon 34134, Republic of Korea

^2^Advanced Photonics Research Institute (APRI), Gwangju Institute of Science and Technology (GIST), Gwangju 61005, Republic of Korea

^3^Department of Physics and KI for the NanoCentury, Korea Advanced Institute of Science and Technology (KAIST) Daejeon 34141, Republic of Korea

^4^Institute of Quantum Systems (IQS), Chungnam National University, Daejeon 34134, Republic of Korea

^†^These authors contributed equally to this work.

^*^*vsepr@gist.ac.kr and ydjang@cnu.ac.kr*

**Fig. S1.** Size distribution of core−shell CsPb(Br_0.2_I_0.8_)_3_@SiO_2_ QDs.


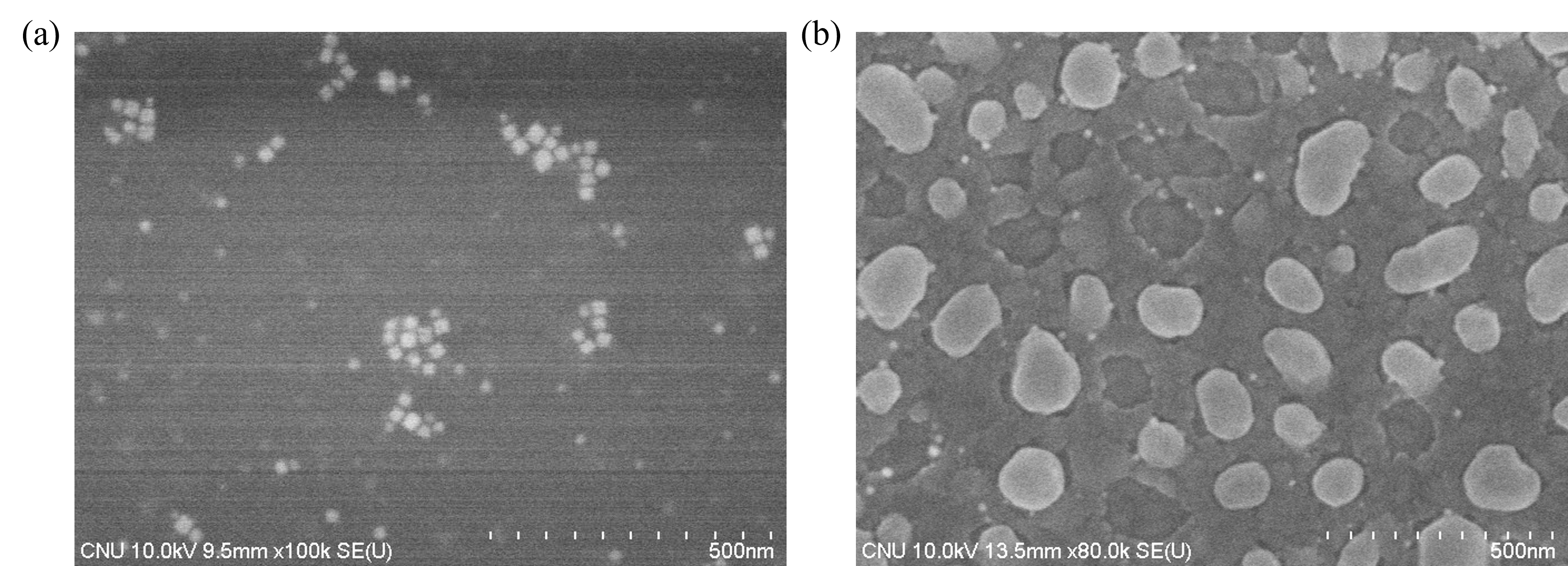


**Fig. S2.** The SEM images of (a) core−shell CsPb(Br_0.2_I_0.8_)_3_@SiO_2_ QDs on SiO_2_ (QDSiO_2_) and (b) core−shell CsPb(Br_0.2_I_0.8_)_3_@SiO_2_ QDs in the hole/sphere–based plasmonic nanogap structure (QDNG). The average density of PQDs is approximately 4–6 PQDs within a 100 nm x 100 nm area.
